# Building Indigenous health workforce capacity and capability through leadership – the Miwatj health leadership model

**DOI:** 10.1017/S1463423621000554

**Published:** 2021-10-07

**Authors:** Stephen Harfield, Carol Davy, Anna Dawson, Eddie Mulholland, Annette Braunack-Mayer, Alex Brown

**Affiliations:** 1 Wardliparingga Aboriginal Health Equity, South Australian Health and Medical Research Institute, Adelaide, South Australia, Australia; 2 School of Public Health, The University of Adelaide, Adelaide, South Australia, Australia; 3 UQ Poche Centre for Indigenous Health, The University of Queensland, St Lucia, Queensland, Australia; 4 Miwatj Aboriginal Health Service, Nhulunbuy, Northern Territory, Australia; 5 School of Health and Society, University of Wollongong, Wollongong, New South Wales, Australia; 6 Adelaide Health Technology Assessment, The University of Adelaide, Adelaide, South Australia, Australia; 7 Adelaide Medical School, Faculty of Health and Medical Sciences, The University of Adelaide, Adelaide, South Australia, Australia

**Keywords:** Aboriginal, Australia, Indigenous, leadership, primary health care, workforce

## Abstract

**Aim:**

In the crowded field of leadership research, Indigenous leadership remains under-researched. This article explores the Leadership Model of an Aboriginal Community Controlled Primary Health Care Organisation providing services to the Yolngu people of remote northern Australia: the Miwatj Health Aboriginal Corporation (Miwatj).

**Background:**

The limited research which does exist on Indigenous leadership points to unique challenges for Indigenous leaders. These challenges relate to fostering self-determination in their communities, managing significant community expectations, and navigating a path between culturally divergent approaches to management and leadership.

**Methods:**

Guided by Indigenous methodology and using a mixed methods approach, semi-structured interviews, self-reported health service data, organisational and publicly available documents, and literature were analysed using a framework method of thematic analysis to identify key themes of the Miwatj Leadership Model.

**Findings:**

The Miwatj Leadership Model is underpinned by three distinctive elements: it offers Yolngu people employment opportunities; it supports staff who want to move into leadership positions and provides capacity building through certificates and diplomas; and it provides for the physical, emotional, and cultural wellbeing of all Yolngu staff. Furthermore, the model respects traditional Yolngu forms of authority and empowers the community to develop, manage and sustain their own health. The Miwatj Leadership Model has been successful in providing formal pathways to support Indigenous staff to take on leadership roles, and has improved the accessibility and acceptability of health care services as a result of Yolngu employment and improved cultural safety.

**Conclusions:**

Translating the Miwatj Leadership Model into other health services will require considerable thought and commitment. The Miwatj Leadership Model can be adapted to meet the needs of other health care services in consideration of the unique context within which they operate. This study has demonstrated the importance of having a formal leadership model that promotes recruitment, retention, and career progression for Indigenous staff.

## Introduction

In Australia, Aboriginal Community Controlled Health Organisations (ACCHOs) have been providing comprehensive and culturally safe primary health care to Aboriginal and Torres Strait Islander communities since 1971 (National Aboriginal Community Controlled Health Organisation; Tilton and Thomas, 2011). In 2016, ACCHOs provided services to more than 370 000 people and employed over 6500 full time equivalent staff (Australian Institute of Health and Welfare, 2018). Just over half of these staff identify as Aboriginal and or Torres Strait Islander peoples (from here on respectfully referred to as Indigenous), making ACCHOs a significant employer of Indigenous peoples in Australia (Australian Institute of Health and Welfare, 2018).

The value of Indigenous health staff within this sector has a positive and powerful impact, not only in terms of acceptability and accessibility of health care but also the role Indigenous health staff play in facilitating cultural safety, defined as ‘Cultural safety is determined by Aboriginal and Torres Strait Islander individuals, families, and communities. Culturally safe practice is the ongoing critical reflection of health practitioner knowledge, skills, attitudes, practising behaviours, and power differentials in delivering safe, accessible, and responsive health care free of racism’ (Australian Health Practitioner Regulation Agency, 2020); and providing leadership and advocating on behalf of their communities (Ware, 2013; Watson *et al.*, 2013; Davy *et al.*, 2016; Harfield *et al.*, 2018). Development of emerging Indigenous leaders is insufficient, however, with an alarming 1 in 3 (33%) Indigenous health staff reporting poor or very poor career development opportunities despite their motivation and commitment to improving the wellbeing and health of their communities (Bailey *et al.*, 2020).

There is a paucity of research exploring Indigenous leadership models internationally, (Evans and Sinclair, 2016), and studies of leadership development in Indigenous primary health care workforce are lacking. This is despite longstanding knowledge of the challenges of recruiting and retaining Indigenous leaders (Hill *et al.*, 2001), which includes operating in two worlds, the Indigenous and Western; the different management and governance structures; and community expectations and commitment. The research which does exist points to unique challenges for Indigenous leaders related to fostering self-determination in their communities, managing community expectations, and navigating a path between culturally divergent approaches to management and leadership (Julien *et al.*, 2010). Indigenous leaders respond to these challenges by both adapting to the contemporary environment and drawing strength from traditional values and approaches. First Nations’ leaders in Canada, for example, ground their leadership in spirituality, use stories for effective communication, draw on the past to inform decisions in the present, and take a holistic approach to decision making (Julien *et al.*, 2010). Likewise, Māori leadership has been described as ‘communication, collaboration and connection’ (p. 311), underpinned by Māori values which are focused around relationships of respect and hospitality with others, interdependence, respect for the spiritual relationship to the gods and cosmos, and responsibility for the environment (Ruwhiu and Elkin, 2016).

To shed light on leadership development models for Indigenous health workforce in primary health care settings, we examined the Leadership Model of the Miwatj Health Aboriginal Corporation in remote Australia. Our aim was to elucidate the elements of the model, the context within which it operates, its benefits, and the challenges to its implementation and ongoing sustainability.

## Methods

**Figure 1.**
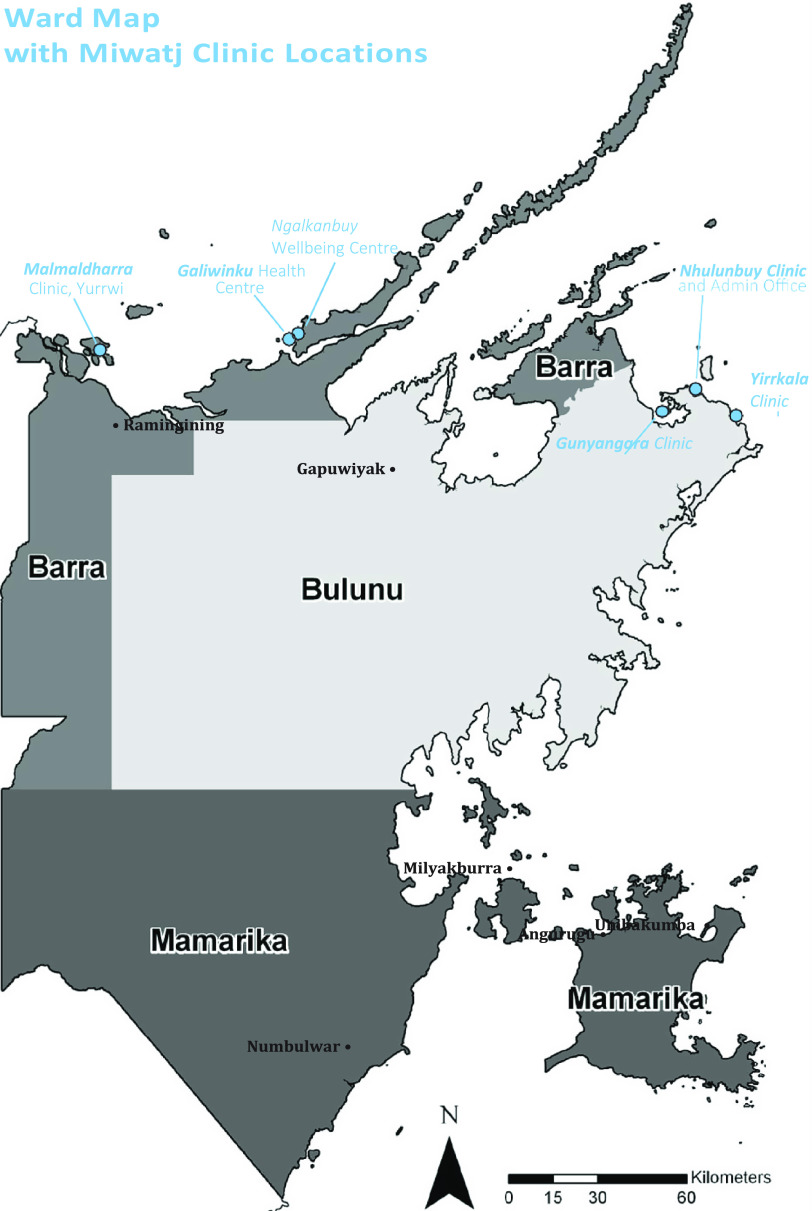
Map of East Arnhem Land and location of Miwatj Health Aboriginal Corporation clinics, 2016.

Miwatj is situated on the Country of the Yolngu (*Yolngu matha* (tongue) word for Aboriginal people) people, the Aboriginal people of East Arnhem Land in north eastern Australia. For many Yolngu people, their languages, lore, and social structures remain absolutely central to their lives. Miwatj was established in 1992 and the Nhulunbuy clinic which houses both administrative and clinical services (located over 1000 km by road east of the nearest capital city Darwin) was erected in 1997. Health care clinics are also located in Gunyangara (also known as Marngarr, Ski Beach), Yirrkala which is approximately 18 km south east of Nhulunbuy, Galiwin’ku (situated on Elcho Island), and Yurrwi (Milingimbi Island) (Figure 1) (Miwatj Health Aboriginal Corporation, 2013). The organisation is committed to ensuring that Yolngu people control the types of and way in which services are provided.

Miwatj Health’s mission is to improve the health and wellbeing of residents of the communities of East Arnhem Land through the delivery of appropriate and comprehensive primary health care and to promote the control by Aboriginal communities of primary health care resources.(Miwatj Health Aboriginal Corporation, 2013: 6).

Miwatj is a large organisation in comparison to other ACCHO (Australian Institute of Health and Welfare, 2018). In the 2016/17 financial year, Miwatj services provided just over 77 000 episodes of care across a range of services and programs: general medical services, prevention and health promotion, social and emotional wellbeing, maternal and child health; chronic disease, pharmaceutical services, visiting medical and allied health specialists, and referrals to secondary and tertiary health services in the Northern Territory, to over 7100 people (Miwatj Health Aboriginal Corporation, 2017). It employed 164 staff, 84 of whom identified as Aboriginal and Torres Strait Islander persons. Most employees were in clinical roles, followed by administration and management (Miwatj Health Aboriginal Corporation, 2017).

As an ACCHO, Miwatj is governed by a Board of Management composed of local Yolngu community members. Miwatj’s governance structure is a reflection of the way in which Yolngu people have always selected leaders. Yolngu leaders are chosen by communities because they demonstrate *‘ngayangu wanggany*’ (one feeling) and ‘*mulkurr wanggany*’ (one mind): they think and feel the same way as the people they lead. Leadership is conditional, constantly earned, and dependent upon their knowledge and ability to support their community (Morphy, 2007).

This study is part of a larger body of research exploring best practice in ACCHOs. Both these studies, and the research program of which it is a part, the Centre of Research Excellence in Aboriginal Chronic Disease Knowledge Translation and Exchange (CREATE), are guided by Indigenous methodology (Wilson, 2008; Smith, 2012). The CREATE research program was defined and guided by a national Leadership Group comprising Aboriginal and Torres Strait Islander and non-Indigenous staff from the ACCHO sector. The Leadership Group identified priority areas for investigation, such as workforce, leadership, governance, accreditation, and funding; and how they contribute to the ACCHO service delivery model and to improving the health and wellbeing of Aboriginal and Torres Strait Islander people. The research was led by an Aboriginal chief investigator, conducted by Aboriginal and Torres Strait Islander and non-Indigenous researchers, and it centred and privileged Indigenous knowledges and values and prioritised the needs of ACCHOs and communities. ACCHOs involved in the study had control over their data, and how it was used and presented.

Prior to the involvement of Miwatj in the study, researchers met with senior Miwatj staff to describe the aims of the research and the nature of data collection, analysis, and reporting. A memorandum of understanding between Miwatj and the South Australian Health and Medical Research Institute (SAHMRI) (research institute), detailing the agreed terms and conditions for undertaking the research, was agreed to before commencement of the study.

We used a mixed-methods approach with semi-structured interviews, self-reported service data, and collection of organisational documents. In addition, we undertook a rapid review of publicly available documents to build an understanding of the local and broader contexts within which Miwatj operates.

Two researchers, one Aboriginal and one non-Indigenous, interviewed staff members at two Miwatj Health sites (Nhulunbuy and Galiwiwin’ku on Elcho Island) between May and September 2016. The Miwatj Chief Executive Office (CEO) and other senior Miwatj staff identified staff to participate. We provided these staff with an information sheet and consent form for consideration and invited them to participate in an interview at a convenient time. We conducted semi-structured interviews face-to-face and recorded them with the participants’ consent.

The participants had the opportunity to review their transcripts and provide corrections. All interview transcripts, organisational documents, and retrieved documents were analysed using a qualitative software package (QSR International Pty Ltd. Version 11). Self-reported service data were collected using a bespoke Case Study Tool [Supplementary File], which ACCHO staff from human resources, financing, and clinical services completed. Data were analysed using a framework method of thematic analysis (Ritchie *et al.*, 2014). The research team developed the analysis framework (Table 1) to initially code deidentified interview transcripts and documents and then met to consider and agree upon the interpretation of the key themes within each of the seven framework components. A draft case study report compiled by the research team was then reviewed and approved by the executive of Miwatj. The research team returned to the organisation at a later date to present the findings to Miwatj Health staff. All research data were stored on password protected servers and restricted to research team members.

**Table 1.** Thematic analysis coding framework

A description of the communities served by the ACCHO and the organisational context within which the **aspect of best practice** operates and or implemented, including the following: • An historical overview • The current environment within which ACCHO operates • A description of the ACCHO
The description of the **aspect of best practice** including the following: • What does it consist of? • When was it developed/implemented? • Why was it developed/implemented? • How has it changed overtime? • Who is it for?
The contributing factors which were considered necessary for the **aspect of best practice** to be implemented and sustained including the following: • Organisational culture • Internal policies and practices • Community members • External partners
The challenges associated with implementing and maintaining the **aspect of best practice** including the following: • Staff attitudes • Community expectations • Resource implications
The types of outcomes resulting from the **aspect of best practice** including any of the following: • Health outcomes • Health practices • Accessibility (acceptability, affordability, awareness etc) for community members • Community participation etc
Recommendations for maintaining and building upon the **aspect of best practice**
Implications for other ACCHOs who may wish to consider developing their own **aspect of best practice**

This study was approved by Menzies School of Health Research Human Research Ethics Committee (HREC 2015-2481), Northern Territory; Aboriginal Health Research Ethics Committee (04-16-651), and University of Adelaide Human Research Ethics Committee (H-2015-221), South Australia.

## Results

Fourteen interviews were completed with 17 staff members, eleven of these participants identifying as Yolngu, one identifying as Aboriginal and Torres Strait Islander and five non-Indigenous. Staff were from two of Miwatj’s sites, seven clinical and two administrative staff members from Galiwin’ku (Elcho Island) and five managers non-clinical, two clinical, and one administrative staff members from Nhulunbuy (Table 2). All participants agreed to have their interviews audio recorded. Four of the participants took the opportunity to review their transcript and to provide additional feedback which was incorporated into the transcripts.

**Table 2.** Interview participant demographics, Miwatj health aboriginal corporation

Participants	Nhulunbuy	Galiwin’ku
Manager – non-clinical		
Female	4	–
Male	1	–
Clinical		
Female	1	6
Male	1	1
Administrative		
Female	1	–
Male	–	2
**Totals**	**8**	**9**

### The Miwatj leadership model

The Miwatj Leadership Model is based on the premise that strengthening the capacity of Yolngu people will better meet the aims and objectives of Miwatj (Miwatj Health Aboriginal Corporation, 2013). The model respects traditional forms of authority and empowers the community to develop, manage, and sustain their own health (Miwatj Health Aboriginal Corporation, 2013). The model spans recruiting Yolngu people into the organisation, developing Yolngu staff who wish to take up leadership roles, and appointing and supporting staff in leadership positions.

### Development of the Miwatj leadership model

There were two key factors in the development of the Miwatj Leadership Model. First, the model was grounded in local initiatives which had been successful in one site of the organisation. The idea of recruiting Yolngu people to leadership positions began at the Galiwin’ku clinic in 2008 as a way to assess local needs and encourage people to engage with the health care service. The community had a vision for Yolngu people to represent their community and to be a voice for the community with the health service. This motivation came from the community:

The push came from the community, they wanted their own people working in the health service. They wanted their own people to do the jobs. So, they could feel, so that the community could feel that they had somewhere to go to and they had someone who knew them, someone who could understand. [Yolngu Clinical]

The community was clear that employing Yolngu staff benefited both the health service and the Galiwin’ku community. Yolngu staff at the time understood and were able to convey the needs of their communities to non-Indigenous colleagues and Miwatji management. This led to an increase in the employment of Yolngu staff at the Galiwin’ku clinic in both clinical and non-clinical roles.

Second, in 2012 discussions were initiated by senior Mitwaj staff to embed the Galiwin’ku model into the Nhulunbuy clinic and administration/management office in Nhulunbuy, as word had spread among the community about the Galiwin’ku model. One of the first steps was to identify key clinical, non-clinical, and leadership positions that had previously been filled by non-Indigenous staff and to review the organisation structure to reflect Miwatj’s mission. This involved a review of workforce procedures within Miwatj:

There was a number of processes… doing the skills audit, looking at the job descriptions and seeing how those skills audits and job descriptions match, looking at ensuring there was some consistency in the bands of the positions… ensure that there was consistency across the board. [Non-Indigenous Manager Non-clinical]

Senior management and the Board of Management who governed Miwatj undertook a change management process to inform all Miwatj staff about the proposed model. Existing policies and procedures were also reviewed, and, where necessary, new ones were developed to ensure there were no systematic barriers to Yolngu staff moving into leadership positions.

The entry pathway for people was important, for all roles. And then looking at the progression structure for people, and then ensuring that there was consistency, and a formal leadership structure within the organisation that’s formally recognised and acknowledged that that’s what it is. [Non-Indigenous Manager Non-clinical]

A selection process to identify Yolngu staff who might be suitable for leadership positions was developed, with two key criteria: potential clinical or administrative capacity and credibility within the community.

So even when we’re recruiting to these positions, we have to consider that we have full representation from all communities around – within this region. And Elders within the community are also consulted, in regard to any new recruits that, you know, people who we’re considering taking on board, to make sure that, you know, they’ve got approval from the Elders. [Yolngu Manager Non-clinical]

Additionally, a leadership incentive scheme was also introduced, to recognise varying levels of ability and the need to renumerate staff appropriately as they continued to build their leadership skills. Staff discussed what leadership was within the context of Miwatj, the different levels of leadership, and how leadership would be demonstrated within each role.

So there are these three different levels here, so we talked about what a beginning leader would need to accomplish and what an intermediate person could do and what an accomplished team leader could do, and depending on where they were at, they’d come in at one of these levels. [Non-Indigenous Manager Non-clinical]

### The Miwatj leadership model today

The Miwatj Leadership Model today is underpinned by three distinctive elements: it offers Yolngu people employment opportunities; it supports staff who want to move into leadership positions; and it provides for the physical, emotional, and cultural wellbeing of all Yolngu staff.

First and foremost, the Miwatj Leadership Model has increased employment opportunities for Yolngu people across the entire organisation. A key element to improving employment of Yolngu people was reviewing Miwatji’s strategic plan and including Yolngu workforce as one of the key performance indicators (KPIs).

The second objective of the Miwatj Leadership Model has been to provide Yolngu staff with opportunities to move into leadership positions. Pathways have been created for these leadership positions to ensure there are no barriers to Yolngu staff fulfilling those roles. The CEO and Human Resources Manager provide whatever support is needed to ensure that local Yolngu people can fulfil their roles. Support includes capacity strengthening initiatives that focus on both leadership qualities and technical skills relevant to the leaders’ roles, and it also evaluates the Yolngu staff member’s existing skills and knowledge as they progress into leadership positions. Previous initiatives in the realm include vocational courses such as certificate-level qualifications in social and emotional wellbeing, and a diploma of management.

There is also an explicit focus on succession planning and supporting future leaders. Promising staff are rotated through various roles within the organisation, with senior members of the team acting as mentors along the way (Lacy, 2014). For example, one Yolngu staff member has acted in several senior management positions including in the CEO role. This opportunity has provided the staff member with senior management experience and has led to them being identified as a role model and someone from whom other staff both Yolngu and non-Indigenous staff can seek advice.

Third, Miwatj has implemented practical initiatives to support the physical, emotional, and cultural wellbeing of Yolngu staff. For example, special leave is available when Yolngu staff need to attend to cultural obligations. Debriefing sessions which allow Yolngu staff to share their concerns and support each other are also encouraged. Grievance procedures have been implemented to allow staff to express and have their issue or concern addressed, either with their direct manager or with more senior management. Also, there are events to help rejuvenate the spirit and culture of Yolngu staff members.

[W]e do have our own sort of cultural practices as well, that can assist that sort of thing. Like healing ceremonies and our camping trips are a good opportunity for the staff to, sort of, re-energise. It’s something that they love doing, you know, reconnecting with the Country, even though the wellbeing camps are focused on whoever our target group is, it’s also a good opportunity for them to, sort of, relax and let their hair down and get away from, sort of, the community pressures and go out for a couple of nights and enjoy reconnecting with land and family. [Yolngu Manager Non-clinical]

### Outcomes of the Miwatj leadership model

#### Employment

**Figure 2.**
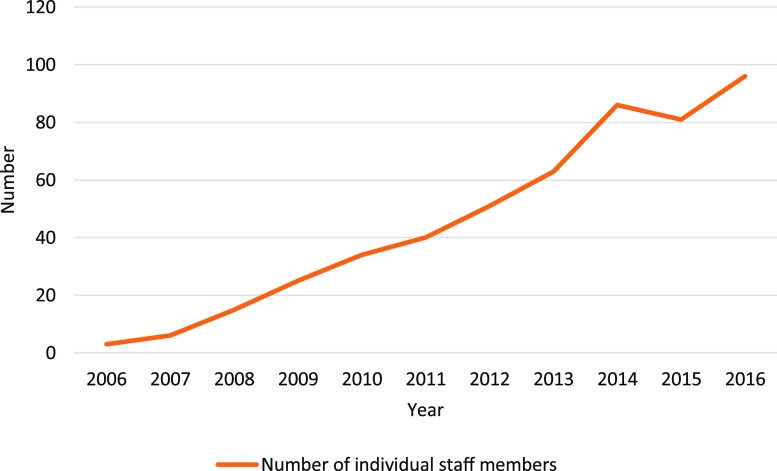
Number of individual Yolngu/Aboriginal staff members employed at Miwatj Aboriginal Health Service, 2006–2016.

The Miwatj Leadership Model has been successful in increasing Yolngu employment and leadership within the organisation. Yolngu staff now lead many of the Miwatj clinical and non-clinical programs and most of the administrative areas (Figure 2). These leaders design and run their own health programs and supervise other Yolngu and non-Indigenous staff, with many of the people appointed to leadership roles also leaders within their own communities. The Miwatj Leadership Model embodies the traditional Yolngu cultural beliefs and values of leadership.

The benefits extend beyond the growth in Yolngu employment and leadership. Miwatj is now considered an employer of choice for Yolngu people; it is a place where Yolngu people now want to work. Additionally, Yolngu staff, including those who do not move into leadership positions, have more meaningful and satisfying employment opportunities. In interviews, Yolngu staff reported gaining a great deal of personal satisfaction from knowing that they have been able to directly contribute to the health and wellbeing of their communities: ‘it’s personal for me anyway’ [Yolngu Manager Non-clinical] and ‘it’s about the bigger picture’ [Aboriginal Manager Non-clinical].

The Miwatj Leadership Model has also contributed to a more stable workforce. In the past, the delivery of services had been jeopardised by a high turnover of non-Indigenous staff. However, employing staff from the Yolngu community has stabilised the workforce and ensured that organisational corporate knowledge is maintained.

#### Community empowerment

The Miwatj Leadership Model has led to broader benefits for the Yolngu communities. Specifically, it has enhanced the Yolngu communities’ capacities to determine their own futures. For example, the Board of Management of Miwatj now plays an important a role in determining how the service operates and is delivered, particularly through the recruitment and subsequent appointment of Yolngu staff to leadership positions. Additionally, Yolngu staff and their communities now contribute more effectively to decisions about the types of services Miwatj provides, by whom, and how they are provided.

The Miwatj Leadership Model also strengthens self-determination in Yolngu communities. The developments at Miwatj have led to ‘social transformation through empowerment’ [Aboriginal Manager Non-clinical] by actively promoting self-determination.

You will see that all of [the staff of Miwatj] their kids go to school and if you go to the Yolngu school now there would be say 70–80% more Aboriginal people, Yolngu people, in that school than there would have been 10 years ago, and a lot of it has got to do with we’ve employed people in meaningful jobs, valued what they do in their jobs, and brought people into town, we’ve given them houses. This is all a part of it. [Aboriginal Manager Non-clinical]

#### Service delivery

Participants believed the Miwatj Leadership Model has significantly improved patient outcomes. In particular, the participants argued that the Yolngu leadership model has been the driving force behind Miwatj’s outstanding health performance outcomes. These have included an increase in the proportion of the population receiving adult health checks and General Practitioner Management Plans undertaken by Miwatj, both of which were well above both the Northern Territory and national averages (Miwatj Health Aboriginal Corporation, 2013; Miwatj Health Aboriginal Corporation, 2017). Participants also believed that these (KPIs) reflect tangible improvements in the health of Yolngu people.

What we’re getting is not just health outputs from KPIs, they’re health outcomes as well, things like changes in the normal birth weights of babies, that’s a health outcome, and obviously immunisation, once you immunise someone they’re not going to get any disease that they been immunised against. [Aboriginal Manager Non-clinical]

The staff explained the links between the leadership model and improved health outcomes in two ways. First, they cited improvements in the accessibility of services. Both Yolngu and non-Indigenous staff spoke about the direct relationship between the employment of Yolngu staff and a significant improvement in the number of people accessing Miwatj services.

So Yolngu, I think, you know, the amount of referrals that we do receive from community, proves to us that they’ve accepted what we do and can see that, you know, we’ve had referrals for our team to go and facilitate a family meeting between two clans. [Yolngu Manager Non-clinical]

Furthermore, Yolngu staff have their ‘ears to the ground in the community’ [Non-Indigenous Clinical] to encourage and support individuals who were unable or reluctant to engage with care.

Second, the staff pointed to improvements in cultural safety. Increasing the proportion of staff who are Yolngu has created a better understanding in the organisation of the crucial relationships and kinship concepts which underpin respectful interactions between patients and providers and which can bridge the divide between traditional knowledge and the biomedical model (Miwatj Health Aboriginal Corporation, 2013).

If you’re going to give a really good treatment…. It’s important that we have the right people dealing with them, right kinship, right relationship, […] The Miwatj model – So in the middle you have a biomedical model of health, so everything’s got to be evidence-based…. But it’s woven into a context which is very culturally appropriate, culturally secure. [Non-Indigenous Manager Non-clinical]

The Yolngu community can now receive health care from someone who is from their local community and speaks *Yolngu matha* (Yolngu tongue). They are no longer required to speak in English. ‘[I]t’s really important for Yolngu people to speak in language and explain to [patients] in language’ [Yolngu Clinical & Yolngu Clinical]. Additionally, establishing Yolngu people as leaders who oversee the way in which non-Indigenous staff relate to patients also builds the cultural safety of services at Miwatj.

I worked alongside – there was always an Aboriginal health worker that would work alongside with me…, but to actually be guided, because you need, non-Indigenous need mentoring. Otherwise, non-Indigenous take over and say, ‘We’re going to do it this way.’ …it needs to be, you know, it’s a pathway, it’s two-way. [Yolngu Clinical & Non-Indigenous Clinical]

Simply put, the Miwatj Leadership Model embeds a form of cultural safety for patients that extends well beyond simple sensitivity to the local culture. It fosters a deeper level of interaction and thoughtful practice that ensures safety as Yolngu communities define it (Nash *et al.*, 2006; Coffin, 2007).

### Challenges

Overwhelmingly, the Miwatj Leadership Model is regarded as a success. Of course, though, this success has brought with it a range of challenges for both Yolngu and non-Indigenous staff. The most frequently noted challenged related to the idea that Yolngu staff were on call 24 h a day, seven days a week.

It’s 24/7 for a lot of us, because we’re all related. You know, we’re a local Yolngu from this region and as soon as you’re, sort of, classed as a [Miwatj] worker, there’s more of a high expectation from the community for you to get involved in a lot of the issues that are happening in community. It doesn’t matter what time of day or night it is. [Yolngu Manager Non-clinical]

Being a member of the local community means Yolngu staff are expected to maintain high standards in relation to their conduct at all times. Participants believed that it was important that Yolngu staff were seen to ‘practise what they preach’ [Yolngu Manager Non-clinical]. Community members who believed these high standards had not been met were quick to criticise and ‘they’re [staff] pulled up straight away’ [Yolngu Manager Non-clinical].

Additionally, earning a good salary when other members of the community were clearly struggling to make ends meet also proved challenging for some Yolngu staff. Often younger staff who join as Aboriginal health practitioners or as trainees ‘get a lot of humbug from other family members’ because they are earning good salary [Non-Indigenous Manager Non-clinical]. Younger and less experienced Yolngu staff who had been appointed to leadership positions were particularly susceptible to criticism. The commitment to support younger and less experienced Yolngu staff into leadership roles has therefore been particularly important regardless of their position or stage of their leadership journey.

There have been difficulties for some non-Indigenous staff. Despite the care taken to manage the change process, some non-Indigenous staff had been resentful of Yolngu staff being promoted into leadership roles.

Now, everyone was on board at the beginning, they thought it was a really great idea, everyone was talking the talk, they understood the concept of empowerment, they thought it was a great idea. So, on paper it started to be developed, there were workshops being held, the clinic manager at the time, was involved in that. And then there were a few hiccups along the way because we started to realise that we could actually start to implement it but there was some resistance to it, and I think it was predominantly from the *Balanda* [non-Indigenous] staff. [Non-Indigenous Manager Non-clinical]

The main issue appeared to be that some non-Indigenous people felt that clinical qualifications were a better indicator of leadership ability than community and cultural knowledge. Clearly, overcoming these types of challenges has been difficult for Miwatj, with a small number of non-Indigenous staff members leaving employment.

### Sustaining the Miwatj leadership model

Various factors are contributing to the sustainability of the Miwatj Leadership Model. Participants spoke about the number of Yolngu people living in the region. Two other issues emerged – developing leaders of the future and sufficient training and development funding. The critical mass of Yolngu staff members within Miwatj is critical to the sustainability of the Miwatj Leadership Model. The significant number of Yolngu people living in East Arnhem Land has meant that there is no shortage of potential candidates.

The focus has been on both capacity strengthening to fill current roles and promoting Miwatj as an employer of choice for future generations.

But mostly supporting the kids here, and also going out to the schools to engage with the kids to see that – show them that this is what our work is, this is how – what we do, we’re here to support you to get you through whatever it might be; but also to show a presence to say, ‘Hey, this is a good opportunity for you to come and join us when you’ve completed school’ [Yolngu Manager Non-clinical]

Participants reported that increased funding is required to sustain the Miwatj Leadership Model. While initially the cost of funding the model may be more expensive, participants believed that in the long term the model will reduce both hospitalisations and the need for more acute services. In particular, funding to support local capacity strengthening programs is crucial. Prior to the reintroduction of local health worker training, staff had to travel to Darwin (a 75-minute aeroplane flight or 1,000 km drive from Nhulunbuy, weather and road conditions permitting) which was not only disruptive to work and family life but also came as an additional cost for Miwatj.

Many of the participants were clear that to be sustainable the Miwatj Leadership Model must always be a work in progress. As a result, management continued to identify new ways to support the employment of Yolngu people and move current staff into leadership positions. This is an ongoing process, as is the refinement of the Miwatj Leadership model.

## Discussion

The Miwatj Leadership Model is a deliberate and determined response to health care challenges faced by Yolngu communities. Built upon a successful strategy implemented by the Galiwin’ku health clinic, the Miwatj Leadership Model is a workforce model that employs and strengthens the capacity of local Yolngu people to take on leadership roles within the organisation. The implementation of the Miwatj Leadership Model has obvious benefits for the staff, the organisation, and the community. For Yolngu staff, there is a supportive workforce environment that recognises the unique and valuable skills, knowledge, and experience that they bring to the organisation. For Miwatj, there is an opportunity to reduce reliance on non-Indigenous workers and staff from outside of East Arnhem Land, a challenge for many ACCHOs (Buykx *et al.*, 2010; Russell *et al.*, 2013; Gwynne and Lincoln, 2017). By employing and strengthening the capacity of local Yolngu people to take on leadership roles, costs associated with external recruitment are minimised and continuity of services is enhanced. Communities were the greatest beneficiaries of the Miwatj Leadership Model. Yolngu staff safeguard the cultural safety of the service through their understanding and provision of care that respects and caters for the values, beliefs, and needs of the communities. This is consistent with other studies, which describe the role of Aboriginal and Torres Strait Islander staff in facilitating cultural safety (Health Workforce Australia, 2011; Panaretto *et al.*, 2014; Harfield *et al.*, 2018). Additionally, Yolngu leaders guide non-Indigenous staff in the practice of culturally safe care, as do Aboriginal and Torres Strait Islander staff across ACCHOs (Abbott *et al.*, 2008; Gwynne and Lincoln, 2017; Lai *et al.*, 2018; Harfield *et al.*, 2018). Importantly, Yolngu staff are also able to assist community members to bridge a number of divides – between traditional and biomedical knowledge systems, between *Yolngu matha* and the English language, and between Yolngu and western social systems, an important role of many Aboriginal and Torres Strait Islander health staff (Health Workforce Australia, 2011; Mercer, 2013; Harfield *et al.*, 2018). Additionally, Yolngu leaders are better able to make informed decisions about the delivery of their health care and the health care of their families and communities (Berner, 1992; Freeman *et al.*, 2014).

There is evidence to suggest a link between the implementation of the Miwatj Leadership Model and longer-term health and social impacts. Employing Yolngu staff has, for example, improved the acceptability and accessibility of services for communities. Other studies have reported similar findings, which suggest that employing Aboriginal and Torres Strait Islander staff has a positive impact on the acceptability and accessibility of health services (Freeman *et al.*, 2014; Panaretto *et al.*, 2014; Gibson *et al.*, 2015; Davy *et al.*, 2016; Harfield *et al.*, 2018). Increased acceptability and accessibility has, in turn, contributed to the higher proportion of the populations receiving childhood immunisation, adult health checks, and chronic disease management plans, all of which are well above averages for the Northern Territory (Miwatj Health Aboriginal Corporation, 2013). It is unclear whether the Miwatj Leadership Model has impacted access to quality care for Yolngu peoples in tertiary referral centres in Darwin and beyond, which is a limitation of the study and an area for future research. Another benefit of the Miwatj Leadership Model is that Yolngu staff are role models for their communities. While this has placed a heavy burden on both their work and private lives, the benefits for community members have been significant. Yolngu staff members, and Yolngu leaders in particular, are able to demonstrate the benefits that come from employment and the importance of self-determination, self-management and community control, frequently the motivation for the establishment of many ACCHOs and employment of local Aboriginal and Torres Strait Islander staff (Taylor *et al.*, 2001; Panaretto *et al.*, 2014; Harfield *et al.*, 2018; Jongen *et al.*, 2019). The Miwatj Leadership Model provides an avenue for Yolngu people to directly contribute to the design of, as well as benefit from the services provided by Miwatj. While the Board of Management is responsible for determining the direction of Miwatj, it is the Yolngu staff who ensure that their vision is realised.

Regardless of its success, there are several challenges to sustaining the Miwatj Leadership Model. These primarily relate to some Yolngu staff who discover how difficult it is to be both a health care provider and a member of the communities they serve. Other challenges include criticism from both Yolngu and non-Indigenous staff who question the appropriateness of leaders who do not necessarily have high-level clinical or administrative formal qualifications. While most non-Indigenous staff have been supportive, a few have found it difficult to work within the organisation and have left. Sustainability also depends on maintaining a critical mass of Yolngu staff now and into the future, appropriate funding for training and development initiatives (Dwyer *et al.*, 2009; Harfield *et al.*, 2018; Lai *et al.*, 2018) and ensuring that the model continues to evolve as the environment changes.

Given the diversity between Indigenous primary health care services, translating the Miwatj Leadership Model into other services will require considerable thought. Incorporating the key contextual factors that support or hinder workforce initiatives aimed at employing and providing pathways to leadership for local Indigenous people will be crucial. Miwatj, for instance, has a ready workforce of local Yolngu people due to the isolated nature of the communities they serve. This will not be the case for all ACCHOs. Miwatj prioritised its commitment to invest in the Yolngu community by including it as a strategic priority and key performance indicator and by making the commitment to support the initiative in every possible way. Additionally, having a strong Board of Management and senior management who shared a common vision was fundamental to the success of the Miwatj Leadership Model. The Miwatj Leadership Model should be adapted to meet the needs of each Indigenous primary health care services and the context within which they operate.

There may also be learning for mainstream services, particularly in relation to the benefits that come from employing and supporting local Indigenous people to take up leadership positions. While variations between health care services must be considered, this study has demonstrated the importance of having a formal leadership model that promotes recruitment, retention, and career progression for Indigenous health care staff.

## Conclusion

The Miwatj Leadership Model has provided more than just a mechanism to increase workforce and leadership opportunities, it has strengthened Yolngu people’ self-determination and community control to ensure their community will always have a health service that is acceptable, accessible and, importantly, delivered by Yolngu people.

## Supplementary material

For supplementary material accompanying this paper visit http://dx.doi.org/10.1017/S1463423621000554. click here to view supplementary material
