# Supplementary material for: Building Indigenous health workforce capacity and capability through leadership – the Miwatj health leadership model
Source: Prim Health Care Res Dev. 2021 Oct 7;22:e52. doi: 10.1017/S1463423621000554 (PMC8515491; doi:10.1017/S1463423621000554)
Supplement: Supplementary file 1 [file S1463423621000554sup001.pdf]

Case Study Tool

Date profile completed	d/m/y numerical field	
Interviewer	first name, last name	
Staff interviewed	position	
Unique study code assigned to service		
**1. HEALTH SERVICE FEATURES at end of the 2017/18 (or 2016/17) Financial Year**		**Source**
How long has the service been operating?	☐ <2 years ☐ 2 – 5 years<br>☐ 6 – 10 years ☐ > 10 years	
Service type - Australian Standard Geographical Classification - (ASGC-RA)[18]	☐ Urban ☐ Regional ☐<br>Remote ☐ Very Remote	
Postcodes covered by service		
Geographic description of area covered by service		
Number of Aboriginal and Torres Strait Islander peoples living within this are as at end 2017/18 (or 2016/17) financial year.		
Name of nearest hospital		
Number of kilometres to nearest hospital	numerical	
Client numbers – total on books at end of 2017/18 (or 2016/17) Financial Year	numerical	
Aboriginal and Torres Strait Islander clients – total at end of the 2017/18 (or 2016/17) financial year	numerical	

Percentage of transient Aboriginal clients<br><br>How was this calculated:<br><br>___________________________________	Approximate percentage (%)	
Episodes of care for Aboriginal and Torres Strait Islander clients for the 2017/18 (or 2016/17) financial year	numerical	
Funding received for the 2017/18 (or 2016/17) financial year	Numerical ($s)	
Number of different funding streams accessed for the 2017/18 (or 2016/17) financial year	☐ 1 to 10 ☐ 11 to 20<br><br>☐ 21 to 30 ☐ > 30	
What proportion of your total funding in 2017/18 (or 2016/17) came from Commonwealth and/or State governments (**Government Funding**)?	Approximate percentage (%)	
What proportion of this **Government Funding** in 2017/18 (or 2016/17) came from Medicare (MBS or PBS)?	Approximate percentage (%)	
What proportion of this **Government Funding** in 2017/18 (or 2016/17) came from a State and/or Territory government?	Approximate percentage (%)	
How many reports were produced for external organisations written in the 2017/18 (or 2016/17) financial year?	☐ 1 to 10 ☐ 11 to 20<br><br>☐ 21 to 30 ☐ > 30	
How many grant applications were written in the 2017/18 (or 2016/17) financial year?	☐ 1 to 5 ☐ 6 to 10<br><br>☐ 11 to 15 ☐ > 15	
How many grant applications were successful in the 2017/18 (or 2016/17) financial year?	☐ 1 to 5 ☐ 6 to 10<br><br>☐ 11 to 15 ☐ > 15	

2. Types of Services Available to Patients		Source
General medical services	☐ Yes ☐ No	
Prevention and health promotion	☐ Yes ☐ No	
Social and emotional wellbeing	☐ Yes ☐ No	
Maternal and child health	☐ Yes ☐ No	
Chronic disease	☐ Yes ☐ No	
Pharmaceutical services	☐ Yes ☐ No	

3. Onsight workforce at end of 2016/17 (or 2015/16) Financial Year	Aboriginal and/or Torres Strait Islander Staff		Non-Indigenous Staff		Source
	FTE Equivalent	Number	FTE Equivalent	Number	
Total Workforce					
Aboriginal or Torres Strait Islander Health Practitioner					
Aboriginal or Torres Strait Islander Health Worker					
Aboriginal or Torres Strait Islander Maternal and Infant Care Worker					
Administration					
Aged care worker					
Allied health					
Diabetes educator					
Enrolled nurse					
General Practitioners					
GP Registrars					
Manager					
Registered midwife					
Registered nurse					
SEWB staff (specify) ________________________					
Other clinical (specify) _________________________					

Other non-clinical (specify)<br>______________________					

4. Regular visiting services in the 2016/17 (or 2015/16) Financial Year		If Yes, how many visits occurred in the 2016/17 (or 2015/16) financial year	Source
Alcohol/tobacco worker	☐ Yes ☐ No		
Cardiologist	☐ Yes ☐ No		
Chronic disease nurse	☐ Yes ☐ No		
Counsellor	☐ Yes ☐ No		
Dentist	☐ Yes ☐ No		
Diabetes educator	☐ Yes ☐ No		
Dietician	☐ Yes ☐ No		
Endocrinologist	☐ Yes ☐ No		
General Practitioner	☐ Yes ☐ No		
Mental health nurse	☐ Yes ☐ No		
Mental health worker	☐ Yes ☐ No		
Nephrologist	☐ Yes ☐ No		
Occupational Therapist	☐ Yes ☐ No		
Ophthalmologist or Optometrist	☐ Yes ☐ No		
Paediatrician	☐ Yes ☐ No		
Physiotherapy	☐ Yes ☐ No		

Podiatrist	☐ Yes ☐ No		
Psychiatrist	☐ Yes ☐ No		
Psychologist	☐ Yes ☐ No		
Social worker	☐ Yes ☐ No		
Other (please specify)<br>______________________			

5. Cultural safety/accessibility		Source
Are there separate men and women entrances and spaces?	☐ Always ☐ Sometimes<br>☐ When requested ☐ Never	
Are Traditional Healers accessible within or through the clinic? E.g Ngangkari	☐ Always ☐ Sometimes<br>☐ When requested ☐ Never	
Are Traditional Healers paid for their consultations?	☐ Always ☐ Sometimes<br>☐ When requested ☐ Never	
Are there Cultural leave provision for Aboriginal staff?	☐ Always ☐ Sometimes<br>☐ When requested ☐ Never	
Do patients have access to interpreter services other than Aboriginal and Torres Strait Islander Health Practitioner or Worker?	☐ Always ☐ Sometimes<br>☐ When requested ☐ Never	

Is there cultural safety training and on-going support provided to the onsite staff and visiting workforce?	☐ Always ☐ Sometimes<br>☐ When requested ☐ Never	
Are non-Indigenous staff provided with an Aboriginal or Torres Strait Islander mentor?	☐ Always ☐ Sometimes<br>☐ When requested ☐ Never	
Does the organisation display Aboriginal or Torres Strait Islander artwork?	☐ Yes ☐ No	
Is transport provided to the service for patients?	☐ Always ☐ Sometimes<br>☐ When requested ☐ Never	
Does your service provide transport to other services?	☐ Always ☐ Sometimes<br>☐ When requested ☐ Never	
How many of the onsite staff speak local language/s?	☐ > 2 ☐ 1 to 2 staff<br>☐ None	
Other (please specify)<br>____________________		

6. Community linkages		Source
Are you an Aboriginal Community Controlled Health Service?	☐ Yes ☐ No	
Does your organisation have a Governing Board?	☐ Yes ☐ No	
What percentage of the Governing Board identifies as Aboriginal or Torres Strait Islander?	☐ 100% ☐ > 50%<br>☐ 10 to 50% ☐ < 10%	
How often does the Governing Board meet?	☐ Regularly ☐ Irregularly<br>☐ Never	
Does the Governing Board have input on operational matters – e.g. Staff recruitment?	☐ High input ☐ Some input<br>☐ No input	
Is the Governing Board actively involved in health service strategic planning, priority setting and health service performance?	☐ Always ☐ Sometimes<br>☐ Never	
Does the broader Community participate in health service planning?	☐ Always ☐ Sometimes<br>☐ Never	
Does your service provide community activities as a way of engagement?	☐ Always ☐ Sometimes<br>☐ Never	
Are patients provided with a formal opportunity to assess their satisfaction with health service? e.g. through surveys	☐ Regularly ☐ Sometimes<br>☐ Never	

**7. Staff Support and Human Resources**		**Source**
Are staff roles well defined and reflected in job descriptions	☐ Yes in all cases ☐ For some staff<br>☐ Not at all	
Are staff offered external professional development opportunities?<br>.	☐ Yes in all cases ☐ For some staff<br>☐ Not at all	
Are staff offered in-service training opportunities?	☐ Yes in all cases ☐ For some staff<br>☐ Not at all	
Are there support structures in place for staff?	☐ Yes ☐ Sometimes<br>☐ No	
What is the average time for clinical staff to remain with the service?	☐ >2 years ☐ 1 to 2 yrs<br>☐ < 1 year	
What is the average time for management staff to remain with the service?	☐ >2 years ☐ 1 to 2 yrs<br>☐ < 1 year	
What is the average time for administration and reception staff to remain with the service?	☐ >2 years ☐ 1 to 2 yrs<br>☐ < 1 year	
Were there any unfilled vacancies for more than 2 months during the 2016/17 (or 2015/16) year?	☐ Yes ☐ No	

Are there any positions you find particularly difficult to fill? If so which: ___________________________		

8. Quality improvement		Source
Is your service involved in a formal quality process in the 2016/17 (or 2015/16) financial year?	☐ Yes ☐ No	
Where in the quality improvement cycle is your service currently?	☐ Just started ☐ Mid cycle ☐ Accredited	
Is there senior staff support for quality improvements?	☐ Yes ☐ No	
Does the service use its own data to review clinical practice?	☐ Yes ☐ No	
If so is the service able to move from identifying needs to implementing practice improvements?	☐ Yes all the time ☐ Sometimes ☐ Not at all	
Does the service use its own non-clinical information to review organisational effectiveness?	☐ Yes ☐ No	
If so, is the service able to move from identifying needs to operational improvements?	☐ Yes all the time ☐ Sometimes ☐ Not at all	

9. Systematic follow up			Source
Is there a system for generating and actioning patient recalls and reminders?	☐ Yes	☐ No	
Is there a system in place for following up abnormal pathology and other test results?	☐ Yes	☐ No	
Is there a system to ensure that staff are registered or credentialed?	☐ Yes	☐ No	

10. Continuity of care			Source
Are there clear internal referral pathways?	☐ Yes	☐ No	
Are there clear external referral pathways?	☐ Always	☐ No	
Are staff available to support patients when they need to access mainstream specialist services?	☐ Always<br>☐ Never	☐ Sometimes	
Is the service notified when a patient is discharged from hospital?	☐ Always<br>☐ Never	☐ Sometimes	

11. Patient information systems		Source
Do you have an electronic information system?<br><br>If yes, what is the system?	☐ Yes ☐ No<br><br>__________________________________	
Is there a current electronic list of clients that is regularly reviewed?	☐ Yes ☐ No	
Is there a system for ensuring patient diagnoses are routinely recorded on the client health summary?	☐ Yes ☐ No	
Do new clinical staff receive training on the information system?	☐ Yes ☐ No	

12. CLINICAL DECISION SUPPORT		Source
Are evidence based guidelines availability and accessible electronically?	☐ Yes ☐ No	
Are they used as routine practice?	☐ Yes ☐ Sometimes ☐ No	
Is training/orientation provided to the use of these resources integrated into in-service training?	☐ Yes ☐ Sometimes ☐ No	
Is there GP support for nurses and Aboriginal and Torres Strait Islander Practitioners and Health workers?	☐ Yes ☐ Sometimes ☐ No	
If yes, is this support available on site?	☐ Yes ☐ Sometimes ☐ No	
Is there specialist support for GPs and other clinical staff	☐ Yes ☐ Sometimes ☐ No	
